# A Novel Bidirectional AlGaN/GaN ESD Protection Diode

**DOI:** 10.3390/mi13010135

**Published:** 2022-01-15

**Authors:** Bin Yao, Yijun Shi, Hongyue Wang, Xinbin Xu, Yiqiang Chen, Zhiyuan He, Qingzhong Xiao, Lei Wang, Guoguang Lu, Hao Li, Yun Huang, Bo Zhang

**Affiliations:** 1 School of Electrical and Information Engineering, Hunan University, Changsha 410082, China; yb1002@163.com (B.Y.); hli@hnu.edu.cn (H.L.); 2 The Science and Technology on Reliability Physics and Application of Electronic Component Laboratory, China Electronic, Product Reliability and Environmental Testing Research Institute, Guangzhou 510610, China; wanghongyue@ceprei.com (H.W.); xuxb11@163.com (X.X.); hezhiyuan1988@126.com (Z.H.); xiaoqingzhong@ceprei.com (Q.X.); leiwang@ceprei.com (L.W.); luguoguang@ceprei.com (G.L.); huangyun@ceprei.com (Y.H.); zhangbo@uestc.edu.cn (B.Z.)

**Keywords:** electrostatic discharge, ESD protection diode, GaN HEMT, transmission line pulsing

## Abstract

Despite the superior working properties, GaN-based HEMTs and systems are still confronted with the threat of a transient ESD event, especially for the vulnerable gate structure of the p-GaN or MOS HEMTs. Therefore, there is still an urgent need for a bidirectional ESD protection diode to improve the ESD robustness of a GaN power system. In this study, an AlGaN/GaN ESD protection diode with bidirectional clamp capability was proposed and investigated. Through the combination of two floating gate electrodes and two pF-grade capacitors connected in parallel between anode or cathode electrodes and the adjacent floating gate electrodes (*C*_GA_ (*C*_GC_)), the proposed diode could be triggered by a required voltage and possesses a high secondary breakdown current (*I*_S_) in both forward and reverse transient ESD events. Based on the experimental verification, it was found that the bidirectional triggering voltages (*V*_trig_) and *I*_S_ of the proposed diode were strongly related to *C*_GA_ (*C*_GC_). With *C*_GA_ (*C*_GC_) increasing from 5 pF to 25 pF, *V*_trig_ and *I*_S_ decreased from ~18 V to ~7 V and from ~7 A to ~3 A, respectively. The diode’s high performance demonstrated a good reference for the ESD design of a GaN power system.

## 1. Introduction

Currently, GaN-based high-electron-mobility transistors (HEMTs) have attracted a great deal of research attention in high-power applications, owing to their low specific on-resistance, high breakdown voltage, high switching frequency and, especially, the more convenient integration (just as the GaN-based monolithic integrated circuits (MICs), which are characterized with low parasitic parameters and high performance) [1,2,3,4,5,6]. Despite the superior operation properties, the GaN-based HEMTs and MICs are still confronted with the threat of failure caused by a transient electrostatic discharge (ESD) event, especially for the vulnerable gate structure of the p-GaN HEMTs, metal–oxide–semiconductor (MOS) HEMTs and Schottky-gated GaN-based HEMTs. In some reports [7,8,9,10,11], it was comprehensively demonstrated that the Schottky-gated GaN-based HEMTs can withstand extremely high transient ESD voltages in the drain-to-source, drain-to-gate and gate-to-source conditions. However, things go differently for the p-GaN (or MOS) HEMTs. We comprehensively investigated the ESD robustness of the p-GaN HEMTs in different conditions [12]. In drain-to-source and drain-to-gate conditions, the equivalent human body model (HBM) failure voltage (*V*_HBM_) of the p-GaN HEMTs can meet the industrial standard (2 kV) [13,14]. However, owing to the lack of discharge path in the gate electrode of the p-GaN HEMTs, the devices exhibit poor ESD robustness in the gate-to-source condition, with an equivalent *V*_HBM_ of only 0.2~0.33 kV. E. Canato [15] and Yiqiang Chen [16,17] reported the gate-to-source ESD failure and degradation mechanisms of p-GaN HEMTs, which mainly rely on the trapping effect and device geometry. To improve the gate-to-source ESD robustness for the p-GaN HEMT, Xin et al. reported a unidirectional AlGaN/GaN ESD protection diode based on a self-triggered discharging channel [14]. The diode can be triggered by a required voltage and possesses a high secondary breakdown current in a forward transient ESD event. However, in a reverse transient ESD event, the unidirectional AlGaN/GaN ESD protection diode will be triggered by a very low voltage, which may be not suitable for GaN-based MICs. In some applications, the ESD protection diode needs to be triggered by a required voltage in both forward and reverse transient ESD events [18]. Therefore, there is still a requirement for a bidirectional ESD protection diode to improve the ESD robustness of a GaN power system.

In this study, a novel bidirectional AlGaN/GaN ESD protection diode (AlGaN/GaN B-ESD-PD) was proposed and investigated. Through the combination of two floating gate electrodes and two pF-grade capacitors connected in parallel between the anode or cathode electrodes and the adjacent floating gate electrodes (*C*_GA_ (*C*_GC_)), the proposed AlGaN/GaN B-ESD-PD could be triggered by a required voltage and possesses a high secondary breakdown current in both the forward and reverse transient ESD events. The paper is organized as follows: the structure and mechanism of the proposed AlGaN/GaN B-ESD-PD are presented in Section 2; the results of the bidirectional leakage current and TLP current–voltage characteristics of the proposed AlGaN/GaN B-ESD-PD, as well as the influence of (*C*_GA_ (*C*_GC_)) and the capacitor connected in parallel between two floating gates to the floating ohmic contact (*C*_G1_ (*C*_G2_)), are investigated in Section 3; the conclusions are drawn in Section 4.

## 2. Structure and Mechanism

**Figure 1:**
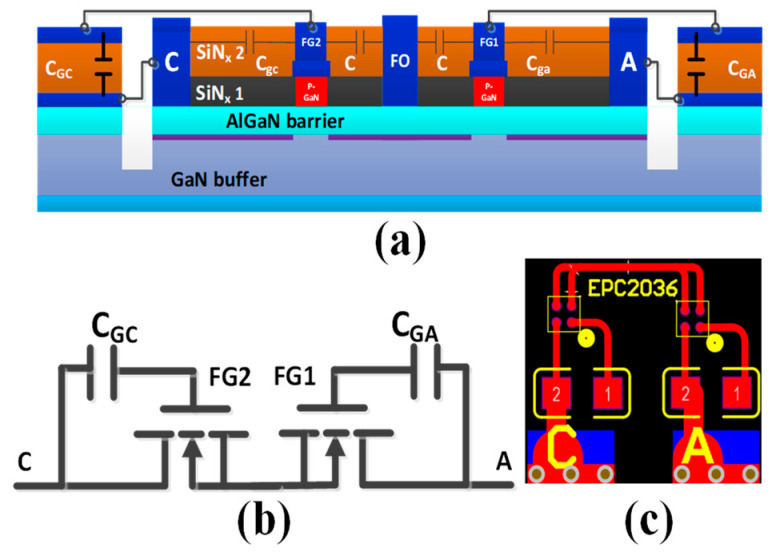
(**a**) The schematic structure and (**b**) equivalent circuit of the proposed AlGaN/GaN B-ESD-PD. (**c**) The equivalent structure configured using the chip capacitor and the commercially p-GaN HEMT (EPC2036) from the EPC Corporation [20].

**Figure 2:**
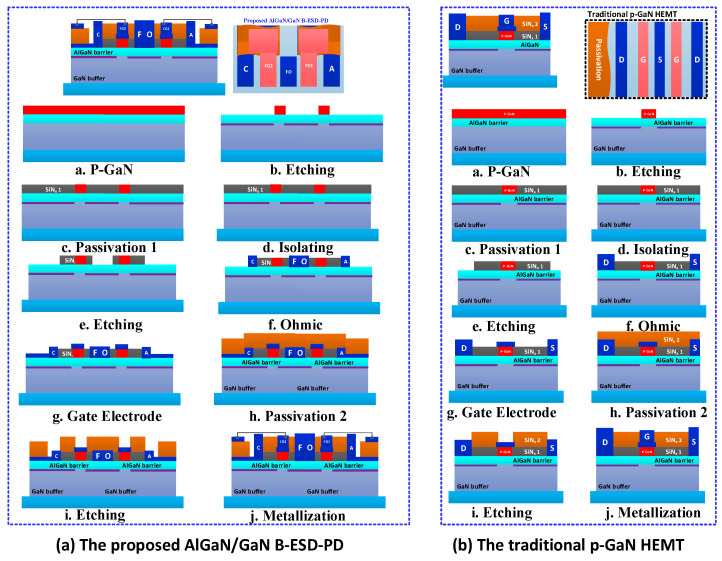
The possible fabrication process of (**a**) the proposed AlGaN/GaN B-ESD-PD and (**b**) traditional p-GaN HEMT.

**Figure 3:**
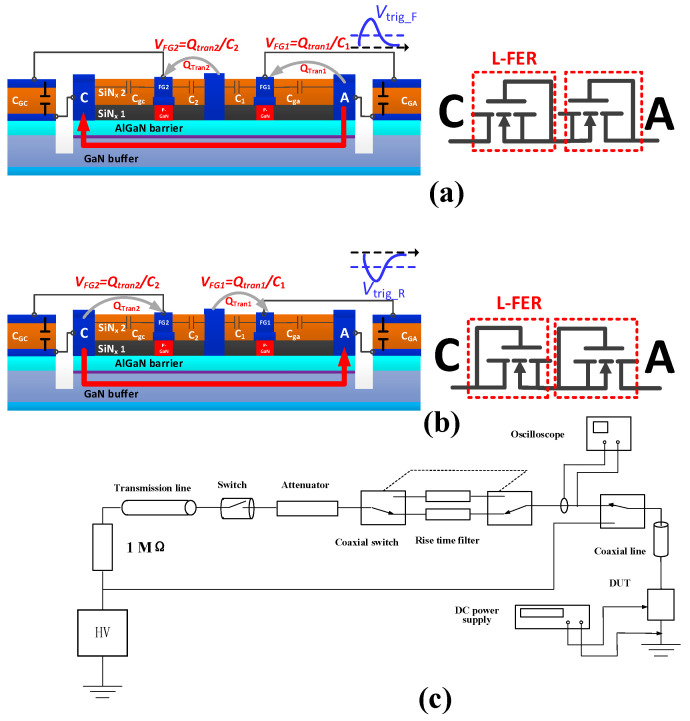
Working mechanism of the proposed AlGaN/GaN B-ESD-PD: (**a**) during a forward transient ESD event, (**b**) during a reverse transient ESD event, and (**c**) the schematic structure for the ESD measurement circuit.

Figure 1a,b shows the schematic structure and equivalent circuit of the proposed AlGaN/GaN B-ESD-PD. The device features two floating gate electrodes (FG1 and FG2), a floating ohmic contact (FO) between the floating gate electrodes, two ohmic contacts as the anode/cathode electrodes (A/C) and two pF-grade capacitors parallelly connected between the anode or cathode electrodes and the adjacent floating gate electrodes (called as *C*_GA_ and *C*_GC_). As it can be seen, the proposed AlGaN/GaN B-ESD-PD is similar to two *E*-mode HEMTs connected in series with the sources tied together. Furthermore, the fabrication process of the proposed AlGaN/GaN B-ESD-PD can be fully compatible with the traditional *E*-mode p-GaN HEMTs (as shown in Figure 2). Therefore, the proposed AlGaN/GaN B-ESD-PD can be easily implemented in state-of-art GaN technology, demonstrating a good reference for the ESD design of the GaN power system. Moreover, the required *C*_GA_ can be easily integrated into the state-of-art GaN technology by changing the area of the capacitor’s metal plate. For example, when the second SiN passivation layer is 100 nm, to obtain a 10 pF capacitor, the required area of the capacitor’s metal plate is 0.0144 mm^2^ (120 μm × 120 μm), which accounts for less than 0.1% of the total area of the traditional p-GaN HEMT in [19]. The working mechanism of the proposed AlGaN/GaN B-ESD-PD is given in Figure 3a,b.

During a forward transient ESD event, a high *dv/dt* can induce a capacitive coupling current from the anode electrode to the cathode electrode (Figure 3a). The capacitive coupling current will carry a certain amount of positive transition charges (*Q*_tran1_ and *Q*_tran2_) to FG1 and FG2, and the positive transition charges will be stored at FG1 and FG2 [12], which can pull down the energy band in the floating gate regions and force the electrons to gather under FG1 and FG2. When the gates’ potentials induced by the positive transition charges exceed the threshold voltage (*V*_th_) of the 2DEG channel, the 2DEG channel under FG1 and FG2 will be turned on. Then, the large current can be passed through the 2DEG channel under FG1 and FG2. Consequently, in a transient ESD event, the proposed structure is similar to two series-connected lateral field-effect rectifiers (L-FER). Therefore, the ESD-event-induced accumulated electrostatic charges can be effectively released through the proposed AlGaN/GaN B-ESD-PD, which can effectively avoid damage to the GaN power system, thereby enhancing the system’s ESD robustness. Similarly, the proposed AlGaN/GaN B-ESD-PD can also effectively avoid damage to the GaN power system during a reverse transient ESD event. Typically, in a forward transient ESD event, the voltage needed to simultaneously turn on the 2DEG channels under FG1 and FG2 (*V*_trig_F_) is positively correlated with *V*_th_ × *C*_1_/(*C*_ga_ + *C*_GA_) and *V*_th_ × (*C*_gc_ + *C*_GC_)/*C*_2_ [12], where *C*_1_ (*C*_2_) is the parasitic capacitance between FG1 (FG2) and FO, and *C*_ga_ (*C*_gc_) is the parasitic capacitance between FG1 and the anode electrode (the cathode electrode). A required *V*_trig_F_ can be obtained by changing *C*_1_ (*C*_2_), *C*_ga_ (*C*_gc_) and *C*_GA_ (*C*_GC_). Similarly, in the reverse transient ESD event, the voltage needed to simultaneously turn on the 2DEG channel under FG1 and FG2 (*V*_trig_R_) is positively correlated with *V*_th_ × *C*_2_/(*C*_gc_ + *C*_GC_) and *V*_th_ × (*C*_ga_ + *C*_GA_)/*C*_1_. A required *V*_trig_R_ can also be obtained by changing *C*_1_(*C*_2_), *C*_ga_ (*C*_gc_) and *C*_GA_ (*C*_GC_).

To reduce the cost of the validation experiment, an equivalent structure configured using the chip capacitors and the commercially p-GaN HEMTs (EPC2036) [20] was used to verify the operating principle of the proposed AlGaN/GaN B-ESD-PD, as shown in Figure 1c. The area of EPC2036 and the required capacitor (as stated above) were only about 0.81 mm^2^ and 0.0144 mm^2^, respectively. Therefore, the total area of the proposed AlGaN/GaN B-ESD-PD was about 1.85 mm^2^, which accounted for less than 5% of the total area of the traditional p-GaN HEMT in [19]. As analyzed above, the ESD protection capability of the proposed AlGaN/GaN B-ESD-PD are related to the continuous working current, threshold voltage and parasitic capacitance between the gate electrode and the drain/source electrode of the commercially p-GaN HEMTs, which were 1.7 A, 2 V and ~10 pF/75 pF, respectively. Moreover, the parasitic capacitances caused in BOEL was less than 1 pF, which did not significantly influence the ESD behavior of the proposed AlGaN/GaN B-ESD-PD. More detailed device characteristics of the commercially p-GaN HEMTs can be found in the datasheet of EPC2036 [20]. In this work, the transient ESD events were produced by our self-developed transmission line pulsing (TLP) measurement system (Figure 3c). The pulse width and rising time in the TLP tests were set to be 100 ns and 2 ns, respectively. To capture the effective transient TLP voltage and current waveforms, the averaged values over the time span from 70% to 90% of the TLP pulse width were extracted. Furthermore, the bidirectional TLP current–voltage (*I–V*) characteristics of the proposed AlGaN/GaN B-ESD-PD were extracted from two of the same devices. The reason for this is explained in Section 3. Moreover, during the TLP test, the sudden obvious decrease in voltage between the anode electrode and cathode electrode was used as a failure criterion.

## 3. Results and Discussion

**Figure 4:**
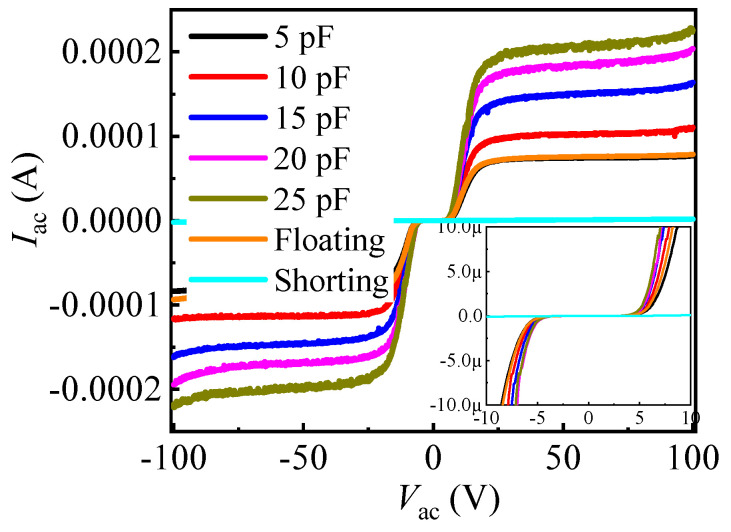
The static bidirectional leakage current characteristics of the proposed AlGaN/GaN B-ESD-PD with different *C*_GA_ (*C*_GC_) values, accompanied by that of the gate-floating and GS-shorting bidirectional GaN diodes.

Figure 4 shows the bidirectional leakage current characteristics of the proposed AlGaN/GaN B-ESD-PD with different *C*_GA_ (*C*_GA_), accompanied by that of the gate-floating bidirectional GaN diode and GS-shorting bidirectional GaN diode. The gate-floating bidirectional GaN diode is similar to two anti-series connected *E*-mode p-GaN HEMTs with two gate electrodes floated, and the GS-shorting bidirectional GaN diode is similar to two anti-series connected *E*-mode p-GaN HEMTs with two gate electrodes shortly connected to the source electrodes. The proposed diodes exhibited a relatively low DC leakage current in different directions; therefore, after the proposed diodes were integrated into the GaN power systems, the forward or reverse DC leakage current of the GaN power systems was not markedly increased. Especially, under the conventional gate working voltage of the traditional p-GaN HEMT (less than 5 V), the DC leakage current of the proposed AlGaN/GaN B-ESD-PD was less than 1 μA. For now, the DC gate leakage current of the traditional p-GaN HEMT was in the range from 20 μA to 320 μA [20]. Among them, the DC gate leakage currents were 160 μA and 320 μA for the devices with static working currents of 30 A and 60 A, respectively. Predictably, the device with higher static working current possessed a higher gate leakage current. Therefore, integrating the proposed AlGaN/GaN B-ESD-PD into the traditional p-GaN HEMT did not obviously increase the DC gate leakage current of the traditional high-current p-GaN HEMT. In addition, as stated above, the fabrication process of the proposed AlGaN/GaN B-ESD-PD can be fully compatible with the traditional E-mode p-GaN HEMTs, making the ESD design more convenient. Although the gate-floating and GS-shorting bidirectional GaN diodes also exhibited a relatively low leakage current in different directions, the diodes were not suitable as the ESD protection diode due to their high triggering voltage (*V*_trig_) and low secondary breakdown current, which is described later.

**Figure 5:**
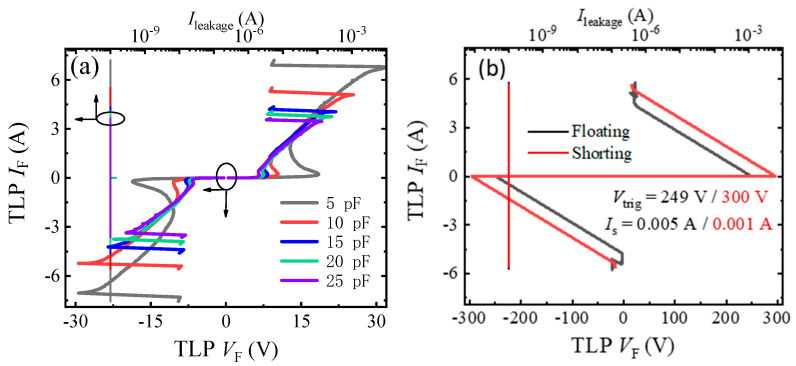
The bidirectional TLP *I–V* characteristics of the proposed AlGaN/GaN B-ESD-PD with different *C*_GA_ (*C*_GC_) values (**a**), and the gate-floating and GS-shorting bidirectional GaN diodes (**b**).

Figure 5 gives the bidirectional TLP current–voltage (*I–V*) characteristics of the proposed AlGaN/GaN B-ESD-PD, with those of the gate-floating and GS-shorting bidirectional GaN diodes shown as references. In both the positive and negative TLP tests, the proposed AlGaN/GaN B-ESD-PD with a *C*_GA_ (*C*_GA_) of 5 pF could be triggered by a voltage of ~18 V and possessed a high secondary breakdown current (*I*_S_) of ~7 A, demonstrating that the proposed diode could effectively release the accumulated electrostatic charges and clamp the potential of the key position to be a required value in both forward and reverse transient ESD events. Therefore, the proposed AlGaN/GaN B-ESD-PD could effectively avoid the ESD damage and enhance the ESD robustness for the GaN power system with the proposed diode integrated. Meanwhile, for the gate-floating and GS-shorting bidirectional GaN diodes, the devices’ triggering voltages (*V*_trig_F_ and *V*_trig_R_) reached about 300 V, and the secondary breakdown currents were as low as 0.01 A. Therefore, in the transient ESD event, the gate-floating and GS-shorting bidirectional GaN diodes could not effectively clamp the potential to be a required value for the key position of the GaN power system, and the low positive secondary breakdown current could not effectively release the accumulated electrostatic charges in the transient ESD event. In other words, the gate-floating and GS-shorting bidirectional GaN diodes may be not suitable as ESD protection diodes to enhance a system’s ESD robustness and to protect the GaN power system from being damaged in a transient ESD event.

**Figure 6:**
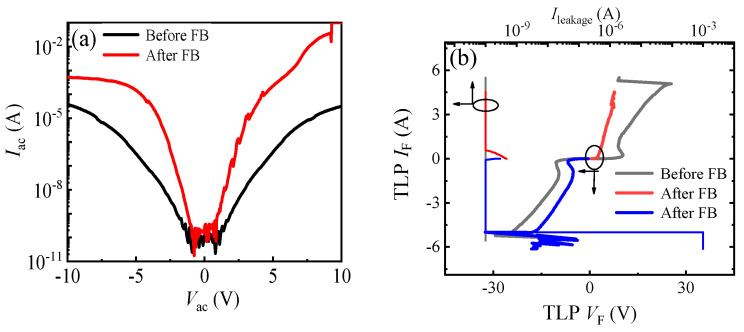
The bidirectional leakage current characteristics on a log-scale (**a**) and TLP *I–V* characteristics (**b**) before and after the forward ESD breakdown. The *C*_GA_ (*C*_GC_) of the proposed AlGaN/GaN B-ESD-PD was 10 pF.

As stated in Section 2, the bidirectional TLP *I–V* characteristics of the proposed AlGaN/GaN B-ESD-PD were extracted from two of the same devices. The reason for that can be explained in Figure 6 in which the bidirectional leakage current characteristics and TLP *I–V* characteristics before and after the occurrence of the forward ESD breakdown (FB) are exhibited. It can be seen that, after the occurrence of the forward ESD breakdown, there was an obvious change in the bidirectional leakage current and TLP *I–V* characteristics for the proposed AlGaN/GaN B-ESD-PD. However, to capture the bidirectional secondary breakdown current of the proposed AlGaN/GaN B-ESD-PD, the device was always tested until the occurrence of an ESD breakdown. Therefore, to obtain the bidirectional TLP *I–V* characteristics of the proposed AlGaN/GaN B-ESD-PD, two of the same devices were needed.

**Figure 7:**
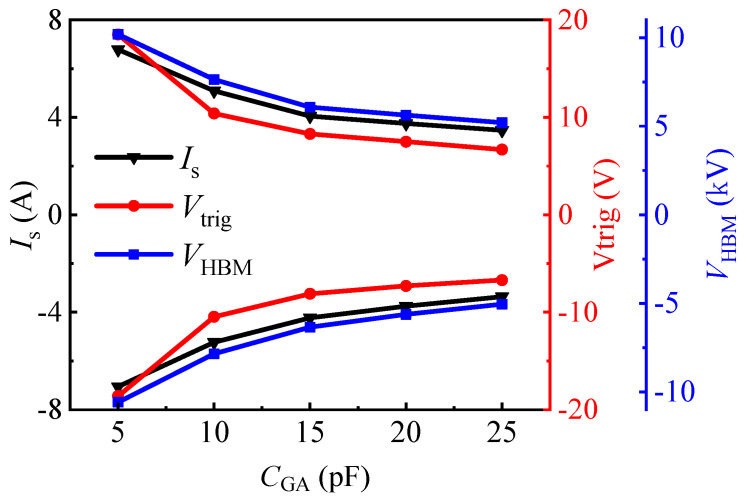
*I*_S_, *V*_trig_ and *V*_HBM_ of the proposed AlGaN/GaN B-ESD-PD with *C*_GA_ (*C*_GC_) increasing from 5 pF to 25 pF.

It can also be seen from Figure 5 that the change in *C*_GA_ (*C*_GC_) had an obvious impact on the bidirectional TLP *I–V* characteristics of the proposed AlGaN/GaN B-ESD-PD. The slope of the snapback region showed a gradual decrease with the increase in capacitor values; this was because increasing the capacitor values decreased the charging rate of the capacitor, which reduced the opening speed of the floating gate structure, subsequently increasing the transient load resistance of the TLP load-line. Furthermore, the influences of *C*_GA_ (*C*_GC_) on the triggering voltage and the secondary breakdown current are summarized in Figure 7. As analyzed above, in the TLP test, the triggering voltages (*V*_trig_F_ and *V*_trig_R_) of the proposed AlGaN/GaN B-ESD-PD were decreased with the increase in *C*_GA_ (*C*_GC_). With *C*_GA_ (*C*_GC_) increasing from 5 pF to 25 pF, the triggering voltages (*V*_trig_F_ and *V*_trig_R_) decreased from 18 V to 7 V. Therefore, through changing *C*_GA_ (*C*_GC_), the desirable triggering voltages (*V*_trig_F_ and *V*_trig_R_) could be obtained for the proposed AlGaN/GaN B-ESD-PD. Similarly, the secondary breakdown currents of the proposed AlGaN/GaN B-ESD-PD were also decreased with the increase in *C*_GA_ (*C*_GC_). With *C*_GA_ (*C*_GC_) increasing from 5 pF to 25 pF, the secondary breakdown currents decreased from 7 A to 3 A. Correspondingly, the equivalent HBM failure voltages (*V*_HBM_ = *I*_S_ × 1500 Ω) of the proposed AlGaN/GaN B-ESD-PD decreased from 10.5 kV to 4.5 kV. The dependence of the secondary breakdown current on the capacitance could have been mainly related to the charging speed of the capacitor. For the proposed AlGaN/GaN B-ESD-PD with a small capacitor, the charging speed of the small capacitor was fast, then the floating gate structure could be opened quickly, and the accumulated electrostatic charges could be quickly released through the proposed AlGaN/GaN B-ESD-PD. As a result, the proposed AlGaN/GaN B-ESD-PD with a small capacitor could release more electrostatic charges and withstand a higher secondary breakdown current. In other words, although changing the *C*_GA_ (*C*_GC_) could regulate the triggering voltages (*V*_trig_F_ and *V*_trig_R_) of the proposed AlGaN/GaN B-ESD-PD, it also weakened the device’s protection capability. Fortunately, even with a high *C*_GA_ (*C*_GC_) of 25 pF, the equivalent HBM failure voltage of the proposed AlGaN/GaN B-ESD-PD could meet the industrial standard (2 kV). Therefore, the proposed AlGaN/GaN B-ESD-PD could serve as an effective ESD protection diode to enhance the system’s ESD robustness and to protect the GaN power system from being damaged in a transient ESD event.

**Figure 8:**
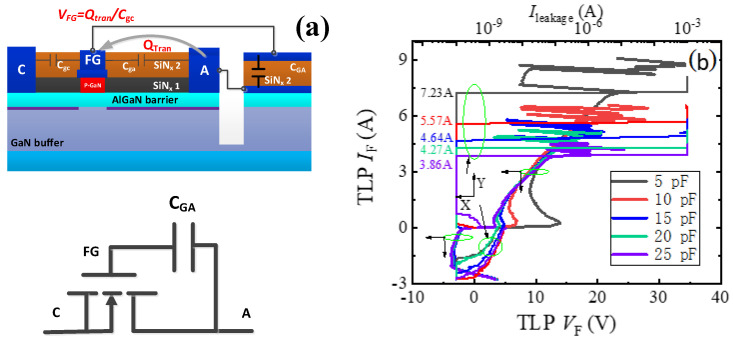
The schematic structure, equivalent circuit (**a**) and bidirectional TLP *I–V* characteristics (**b**) of the unidirectional AlGaN/GaN ESD protection diode.

As stated above, through changing C_GA_ (C_GC_), the desirable triggering voltages (*V*_trig_F_ and *V*_trig_R_) could be obtained for the proposed AlGaN/GaN B-ESD-PD. To make the dependence of the triggering voltage on C_GA_ and C_GC_ clear, the bidirectional TLP *I–V* characteristics of the unidirectional AlGaN/GaN ESD protection diode with different *C*_GA_ values were studied, as shown in Figure 8. The change in *C*_GA_ only had an obvious impact on the positive TLP *I–V* characteristics of the unidirectional AlGaN/GaN ESD protection diode and had no effect on its reverse TLP *I–V* characteristics. In other words, only the forward triggering voltage of the unidirectional AlGaN/GaN ESD protection diode depended on *C*_GA_, and the reverse triggering voltage was not related to *C*_GA_. Because the proposed bidirectional AlGaN/GaN ESD protection diode was similar to two anti-series connected unidirectional AlGaN/GaN ESD protection diodes, it can be inferred that the forward triggering voltage of the proposed bidirectional AlGaN/GaN ESD protection diode is related to C_GA_ and not related to C_GC_, and its reverse triggering voltage is related to C_GC_ and not related to C_GA_.

**Figure 9:**
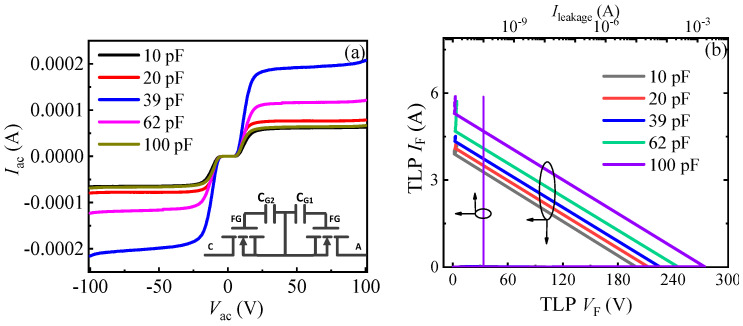
The leakage current characteristics (**a**) and TLP *I–V* characteristics (**b**) of diode 1 with different *C*_G1_ (*C*_G2_) values. The inset of Figure 9a is the equivalent circuit of diode 1.

In order to make a comprehensive comparison, the TLP *I–V* characteristics of the proposed AlGaN/GaN B-ESD-PD with different *C*_G1_ (*C*_G2_) values were also investigated. First, the leakage current and TLP *I–V* characteristics of the two reverse-series connected *E*-mode p-GaN HEMTs with two gate electrodes connected to the floating ohmic electrode through *C*_G1_ and *C*_G2_ are presented in Figure 9. The device is called diode 1 in the following work, and its equivalent circuit is shown in the inset of Figure 9a. As indicated in Figure 8, although diode 1 exhibited a relatively low static leakage current in different directions, the device possessed a high triggering voltage over 200 V and an extremely low positive secondary breakdown current of 0.01 A. In addition, the triggering voltages of diode 1 were increased with the increase in *C*_G1_. Therefore, diode 1 could not effectively clamp the potential to be a required value for the key position of the GaN power system. Moreover, that high triggering voltage is much higher than the safe gate working voltage of the traditional p-GaN HEMT, which will damage the p-GaN gate of the traditional p-GaN HEMT. Furthermore, that low positive secondary breakdown current cannot effectively release the accumulated electrostatic charges in the transient ESD event. On the whole, diode 1 may be not suitable as the ESD protection diode to enhance a system’s ESD robustness and to protect the GaN power system from being damaged in a transient ESD event.

**Figure 10:**
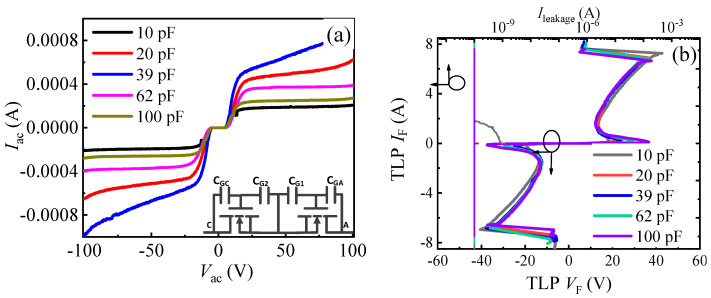
The bidirectional leakage current characteristics (**a**) and TLP *I–V* characteristics (**b**) of diode 2 with different *C*_G1_ (*C*_G2_) values. The inset of Figure 10a is the equivalent circuit of diode 2.

**Figure 11:**
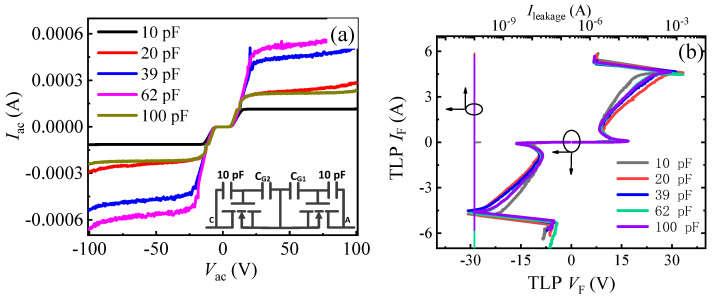
The bidirectional leakage current characteristics (**a**) and TLP *I–V* characteristics (**b**) of diode 3 with different *C*_G1_ (*C*_G2_) values. The inset of Figure 11a is the equivalent circuit of diode 3.

**Figure 12:**
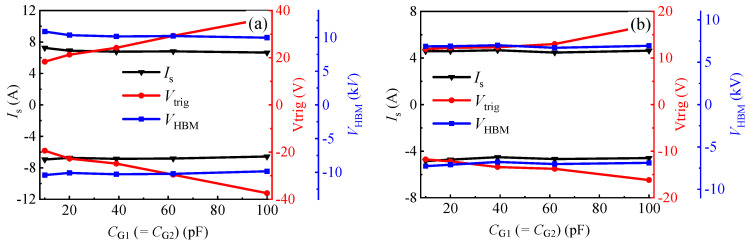
*I*_S_, *V*_trig_ and *V*_HBM_ of diode 2 (**a**) and diode 3 (**b**) with different *C*_G1_ and *C*_G2_ values.

Figure 10 and Figure 11 give the bidirectional leakage current characteristics and TLP *I–V* characteristics of the proposed AlGaN/GaN B-ESD-PD with a *C*_GA_ (*C*_GC_) of 5 pF or 10 pF and different *C*_G1_ (*C*_G2_) values. For simplicity, the diode with a *C*_GA_ (*C*_GC_) of 5 pF was called diode 2, shown in the inset of Figure 10a, and the diode with a *C*_GA_ (*C*_GC_) of 10 pF was called diode 3, shown in the inset of Figure 11a. It is shown in Figure 10 that the triggering voltages and secondary breakdown currents of diode 2 were related to *C*_G1_ (*C*_G2_). With *C*_G1_ (*C*_G2_) increasing from 10 pF to 100 pF, the triggering voltages of diode 2 decreased from 18 V to 36 V, and the secondary breakdown currents slightly decreased from 7 A to 6.5 A. Correspondingly, the equivalent HBM failure voltages of diode 2 decreased from 10.5 kV to 9.75 kV, as summarized in Figure 12a. Therefore, through changing *C*_G1_ (*C*_G2_), the desirable triggering voltages (*V*_trig_F_ and *V*_trig_R_) could also be obtained for diode 2. Meanwhile, for diode 3, the change in *C*_G1_ (*C*_G2_) had nearly no effect on the secondary breakdown currents, only leading to a slight increase in the triggering voltages. Therefore, to obtain desirable triggering voltages (*V*_trig_F_ and *V*_trig_R_) for diode 3, the way of changing *C*_G1_ (*C*_G2_) was not particularly effective. Moreover, compared with diode 2 and diode 3, the proposed AlGaN/GaN B-ESD-PD may be more desired. This is because when using a traditional GaN HEMT, increasing the capacitance should be avoided as much as possible.

## 4. Conclusions

In conclusion, a novel AlGaN/GaN B-ESD-PD featuring two floating gate electrodes and two pF-grade capacitors was proposed for enhancing the ESD robustness of a GaN power system. Through the TLP tests, it was demonstrated that the proposed AlGaN/GaN B-ESD-PD could be triggered by a required voltage (~10 V) and possessed a high secondary breakdown current in both forward and reverse transient ESD events. Furthermore, it was also found that the required triggering voltages and secondary breakdown currents of the proposed AlGaN/GaN B-ESD-PD were strongly related to *C*_GA_ (*C*_GC_). With *C*_GA_ (*C*_GC_) increasing from 5 pF to 25 pF, the positive triggering voltages decreased from 18 V to 7 V, and the positive secondary breakdown currents decreased from ~7 A to ~3 A. In addition, the fabrication process of the proposed AlGaN/GaN B-ESD-PD can be fully compatible with the traditional GaN HEMT, demonstrating a good reference for the ESD design of GaN monolithic integrated circuits.

## Data Availability

Data are available on request due to restrictions, e.g., privacy or ethical restrictions.
